# Frailty in younger adults in hospital

**DOI:** 10.1093/qjmed/hcad173

**Published:** 2023-07-19

**Authors:** E H Gordon, N M Peel, R E Hubbard, N Reid

**Affiliations:** From the Centre for Health Services Research, The University of Queensland, Brisbane, Australia; From the Centre for Health Services Research, The University of Queensland, Brisbane, Australia; From the Centre for Health Services Research, The University of Queensland, Brisbane, Australia; From the Centre for Health Services Research, The University of Queensland, Brisbane, Australia

## Abstract

**Background:**

Even though frailty has been extensively measured in the acute care setting, relatively little is known about the frailty of younger adult inpatients.

**Aim:**

This study aimed to measure frailty in a sample of hospitalized adults aged 18 years and over and to examine how frailty in younger adult inpatients differs from middle-aged and older adult inpatients.

**Design:**

Secondary analyses of prospectively collected cohort data.

**Methods:**

Research nurses assessed 910 patients at admission to four Australian hospitals using the interRAI Acute Care instrument. Comparison of frailty index (FI) scores and domains was conducted across three age groups: younger (18–49 years), middle-aged (50–69 years) and older adults (≥70 years). Multivariable logistic regression examined risk of prolonged length of stay and unfavourable discharge destination.

**Results:**

Younger adults (*n* = 214; 23.5%) had a mean (SD) FI of 0.19 (0.10). Approximately 27% (*n* = 57) of younger adults were frail (FI > 0.25). Mood and behaviour, health symptoms and syndromes, nutrition and pain were the most frequently affected domains in younger adults and 50% had ≥3 comorbidities. Frailty increased the risk of long length of stay (odds ratio (OR) = 1.77, *P* < 0.001) but not the risk of an unfavourable discharge (OR = 1.40, *P* = 0.20) in younger adults.

**Conclusions:**

This study showed that frailty is prevalent in younger patients admitted to acute care and is associated with adverse outcomes. This study was a critical first step towards establishing an understanding of frailty in younger hospitalized adults.

## Introduction

Frailty is a state of increased vulnerability to adverse outcomes.^1^ Even though frailty increases with age and is generally classed as a geriatric syndrome, it is not synonymous with age. In 2011, Rockwood *et al*.^2^ analysed longitudinal data from the National Population Health Survey and calculated a frailty prevalence estimate (using the frailty index (FI)) of 2.4% in adults aged 15–39 years and found that these frail adults were at increased risk of death than their relatively fit counterparts. More recently, using UK Biobank data, Hanlon *et al*.^3^ reported 2–3% frailty prevalence rates for women and men aged 37–45 years and 45–55 years. Frailty was associated with mortality in all groups except women aged 37–45 years. Evidently, even very young adults are not exempt from accumulated deficits that make them vulnerable to poor outcomes.

Even though frailty has been extensively measured in the acute care setting,^4^ relatively little is known about the frailty of younger adult inpatients. Here, we aimed to measure frailty in a sample of hospitalized adults aged 18 years and over and to examine how frailty in younger adult inpatients differs from middle-aged and older adult inpatients.

## Methods

### Study design, setting and participants

A prospective cohort study was conducted in four Australian hospitals in 2015–16. Patients aged 18 and older, admitted to general medicine, surgical, oncology and orthopaedic wards, and mixed wards in the rural hospitals, were eligible to participate.

Ethics approval for the study was obtained from Hospital Human Research Ethics Committees, and patients gave written informed consent to participate. For patients with cognitive impairment, consent was sought from the appropriate substitute decision-makers.

### Data collection

The interRAI Acute Care (AC) instrument^5^ was used to assess patients within 12 h of admission to the ward. This instrument is a shortened version of the interRAI AC-CGA, which was designed to support comprehensive geriatric assessment (CGA) of older people with complex care needs in the acute hospital setting. The interRAI AC supports universal assessment and care planning of all adult acute inpatients, while preserving the important diagnostic and screening functions of the interRAI AC-CGA. The interRAI AC assesses clinical items across multiple psychosocial and functional domains and has good inter-rater reliability. To obtain information for each item, trained research nurses use patient and family interviews, direct observations, staff interview and medical records.

### Measures

Standardized methodology was used to derive a 56-variable FI.^6^ Variables (also termed ‘deficits’) included cognitive function, communication, mood and behaviour, physical function, continence, number of comorbidities, health conditions, nutritional status, skin integrity and number of medications. As per standard convention, the FI was calculated by summing the number of deficits and dividing by the total number of deficits assessed. For patients with missing items, the denominator was reduced accordingly.

Length of stay and discharge destination were identified from medical and administrative records.

### Analysis

Comparisons of categorical variables (chi-square), median length of stay and mean FIs (analysis of variance) were conducted across three age groups: younger (18–49 years), middle-aged (50–69 years) and older adults (≥70 years). The distribution of domains contributing to the FI was also examined by age group and the proportions were compared (Chi-square) across the three age groups. Logistic regression, adjusted for age and sex, was used to examine the associated between increases in the FI (in 0.1 increments) and outcomes including long length of stay (>75th percentile) and adverse discharge outcomes (those who did not return to their usual place of residence after the acute care episodes or who died in acute care).

## Results

Participant characteristics are presented in Table 1. The study population included 910 adults (434 female; 47.7%) aged between 18 and 99 years (median age = 66 years). Two hundred and fourteen (23.5%) of the study population were aged 18–49 years. The majority of younger adults were community-dwelling (98.6%) and approximately one-quarter had an admission in the preceding 30 days (24.9%). There were no in-hospital deaths in this age group; however, 6.5% were not discharged home at the end of their acute admission.

**Table 1.** Participant characteristics

Characteristic	Younger adults	Middle-aged adults	Older adults	*P*-value
	18–49 years	50–69 years	≥70 years	
	*N* = 214	*N* = 303	*N* = 393	
Age, mean (SD)	35.0 (8.9)	60.5 (5.7)	79.5 (6.4)	NA
Female, *n* (%)	110 (51.4)	131 (43.2)	193 (49.1)	0.14
Indigenous status,^a^*n* (%)	10 (4.7)	7 (2.3)	5 (1.8)	0.08
Country of birth not Australia, *n* (%)	51 (23.8)	85 (28.1)	140 (35.6)	0.08
Primary language not English, *n* (%)	16 (7.5)	20 (6.6)	52 (13.2)	0.006
Marital status				
Married/partnered, *n* (%)	112 (52.3)	166 (54.8)	195 (49.6)	0.40
Never married/widowed/separated or divorced, *n* (%)	102 (47.7)	137 (45.2)	198 (50.4)	
Living arrangement at admission				<0.001
Community-Dwelling, *n* (%)	211 (98.6)	299 (98.7)	363 (92.4)	
Institution,^b^*n* (%)	3 (1.4)	4 (1.3)	30 (7.6)	
Lives alone, *n* (%)	18 (8.4)	81 (26.7)	102 (25.9)	<0.001
Does not have a support person for discharge, *n* (%)	4 (1.9)	12 (4.0)	11 (2.8)	<0.001
Recent hospitalization, *n* (%)	53 (24.9)	71 (23.4)	96 (24.5)	0.92
Admitting unit, *n* (%)				
Medical	32 (25.6)	48 (27.9)	78 (39.6)	0.01
Mixed	23 (18.4)	44 (25.6)	47 (23.9)	
Orthopaedic	27 (21.6)	27 (15.7)	25 (12.7)	
Surgical	43 (34.4)	53 (30.8)	47 (23.9)	
FI, mean (SD)	0.19 (0.10)	0.23 (0.11)	0.32 (0.14)	<0.001
FI > 0.40, *n* (%)	8 (3.7)	20 (6.6)	100 (25.4)	
Outcomes				
Length of stay, median (IQR)	2 (1–5)	2 (1–6)	3 (2–7)	0.083
Change of care^c^ or died,^d^*n* (%)	14 (6.5)	16 (5.3)	59 (15.0)	<0.001

FI, frailty index; SD, standard deviation; IQR, interquartile range.

a Indigenous status relates to individuals who identify as Aboriginal and/or Torres Strait Islander.

b Institution refers to RACF, supported living, other hospital.

c Change of care includes those who did not return to usual place of residence on completion of acute care episode but required continuing care (e.g. other acute care setting, palliative care or rehabilitation) or were newly discharged to residential aged care.

d Seven patients died (6 in the ≥70 years age group and 1 in the 50–69 years age group).

**Figure 1.**
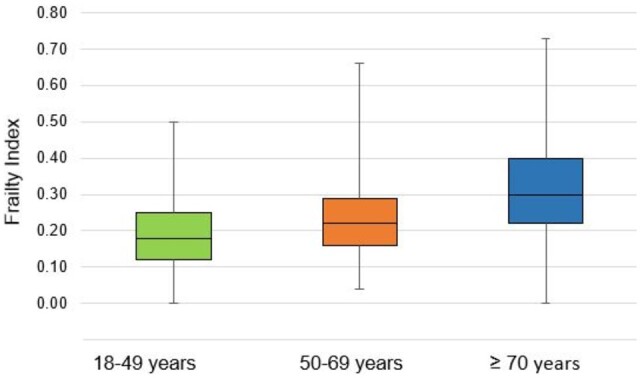
Distribution of the frailty index by age group.

**Figure 2.**
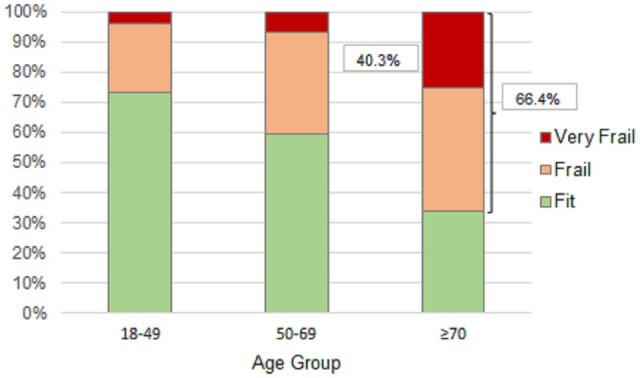
Frailty categories by age group. *Note*: Fit = FI < 0.25, Frail = FI 0.25–0.4; Very Frail FI > 0.40.

The mean FI increased significantly across the age groups, from 0.19 in younger adults (<50 years) to 0.32 in older adults (≥70 years) (*P* < 0.001; Table 1, Figure 1). In the young adult group, the FI ranged from zero to 0.50 (Figure 1; corresponding to zero through to 28 deficits). More than one-quarter (26.6%) were classified as frail (FI = 0.25–0.40 = 22.9%) or very frail (FI > 0.40 = 3.7%; Figure 2).

**Figure 3.**
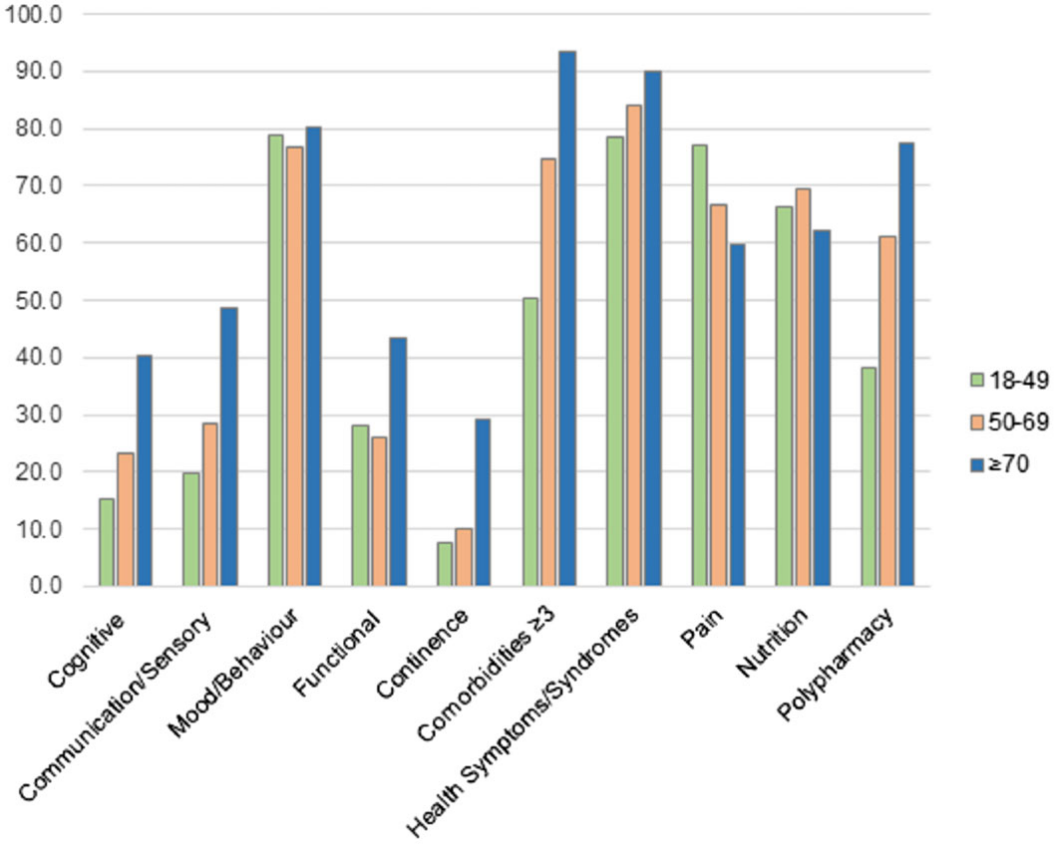
Deficits in frailty index domains by age group.

The young adult group had deficits in all domains (Figure 3). The four most affected domains were mood and behaviour, health symptoms and syndromes (e.g. fatigue, dyspnoea, falls), nutrition, and pain. Fifty percent of younger adults had three or more comorbidities. Between age-group differences were significantly different for all domains (all *P* < 0.001) except mood and behaviour (*P* = 0.469) and nutrition (*P* = 0.137).

In younger adults, after adjustment for age and sex, each 0.1 increment in the FI was associated with 77% higher odds of a prolonged length of stay (odds ratio (OR) = 1.77, 95% confidence interval (CI): 1.27–2.48, *P* < 0.001; Table 2). It was not associated with a higher odds of an unfavourable discharge outcome (OR = 1.40, 95% CI: 0.84–2.32, *P* = 0.20). Increments in the FI were associated with higher odds of prolonged length of stay in middle-aged and older adults but were only associated with higher odds of an unfavourable discharge outcome in older adults (Table 2).

**Table 2.** Multivariable logistic regression: odds of an adverse outcome with every 0.1 increment in the FI

Outcome	Younger adults	Middle-aged adults	Older adults
	18–49 years	50–69 years	≥70 years
Prolonged length of stay^a^	1.77 (95% CI: 1.27–2.48)***	1.39 (95% CI: 1.06–1.82)*	1.19 (95% CI: 1.01–1.40)*
Change of care^b^ or died	1.40 (95% CI: 0.84–2.32)	1.35 (95% CI: 0.86–2.11)	1.31 (95% CI: 1.08–1.60)**

Models adjusted for age and sex.

FI, frailty index.

a >75% percentile.

b Change of care includes those who did not return to usual place of residence on completion of acute care episode but required continuing care (e.g. other acute care setting, palliative care or rehabilitation) or were newly discharged to residential aged care.

* *P* < 0.05,

** *P* < 0.01,

*** *P* < 0.001.

## Discussion

In this study, more than one-quarter of younger inpatients (aged 18–49 years) were frail and some were even severely frail. Younger adults were particularly affected by mood and behavioural symptoms, health symptoms and syndromes, nutrition-related impairments and pain; however, deficits arose in all health domains. No young inpatients died in hospital; however, frailty increased the risk of prolonged hospitalization.

It is difficult to contextualize these results as this is the first study, to our knowledge, to examine frailty in a general inpatient population that includes (very) young adults. In their recent rapid review of frailty in younger populations, Spiers *et al*.^7^ identified 268 studies where the majority of participants were aged 60 years or younger. Less than one-third of study populations had a mean or median age below 50 years and the majority were conducted in clinical subgroups with chronic or life-limiting diseases (such as end-stage organ failure). A proportion of studies were conducted in surgical populations. In a study of emergency general surgical patients,^8^ for example, frailty (as measured by the Clinical Frailty Scale) was present in 1% (8/646) of patients aged <40 years and 5% (32/668) of patients aged 40–59 years. In another study of the same clinical population, the prevalence of frailty was 16% in those aged 40–64 years.^9^ Regardless of age, frailty was associated with poor clinical outcomes including mortality.^8^ These estimates of prevalence are lower than reported in the current study, which may reflect important clinical differences in patient populations, such as higher rates of multimorbidity in general medical patients. In the current study, half of the younger adults had three or more co-morbidities and one-quarter had been recently admitted to hospital.

Using different inclusion criteria, Loecker *et al*.^10^ performed an integrative review of studies of 18–65 year olds. Estimates of frailty prevalence ranged from 3.9% to 63% in community-dwellers with chronic diseases (such as diabetes, human immunodeficiency virus) and social deprivation (such as homelessness). This review did not include any studies of young hospitalized adults, however, it also identified depressive symptoms, abnormal body mass index and pain as key factors associated with frailty in younger populations.

Routine frailty assessment has been investigated and implemented for older community-dwelling adults.^11^ Interventions for frailty have been almost exclusively studied in adults over 65 years and the evidence is mounting that frailty is modifiable.^12^ While it is possible that frailty interventions would improve the balance of health deficits and assets in younger and middle-aged adults, lack of evidence precludes recommendations regarding screening for frailty in young community-dwellers.

It is important to consider whether frailty in young inpatients is, in fact, *acute illness* in young inpatients. The FI used in this study captured deficits at admission, and acute illness and hospitalization may have impacted many (such as activities of daily living and pain) but not all variables (such as vision and hearing impairment and abnormal body mass index). The high rates of co-morbidity and recent hospitalization points to underlying vulnerability in at least a proportion of the group. In older adult populations, those most at risk of hospitalization are frail elders residing in the community or aged care facilities. Future studies may confirm whether baseline frailty is also high in younger inpatients relative to community-dweller norms.

Regardless of whether frailty primarily captures baseline or acute vulnerability, its assessment may play an important role in younger inpatients. It has the potential, for example, to contribute to comprehensive risk assessment, which in turn may influence selection of treatment options and guide shared decision-making, all with view to achieving better outcomes in the short and possibly long term. Frailty in older community-dwellers and inpatients is associated with socioeconomic disadvantage.^13^^,^^14^ It seems likely that social vulnerabilities, such as homelessness, unemployment, low education level and poor health literacy, would also be prevalent in frail younger inpatients. Thus, frailty assessment in hospital may be a key opportunity to intervene and mitigate the progression of frailty into older age through individualized multidisciplinary management.

The strengths of this study include the comprehensive data collection using a standardized instrument from a cohort of inpatients across several medical disciplines and sites. Study limitations include the risk of selection bias related to recruitment being limited to weekdays within 12 h of arrival to an inpatient unit and nonparticipation secondary to acute illness and language barriers. The study sites were located in two Australian states and, as a result, study findings may not be generalizable to other inpatient populations within Australia or internationally. While the study was adequately powered to explore differences in the FI and deficit prevalence rates, future larger studies of younger adult inpatients are required to confirm the relationship between the FI and prolonged hospitalization and to explore the relationship between FI and other adverse outcomes. ‘Unfavourable discharge’ may not be a clinically relevant outcome for younger adults in hospital as institutional care is, in Australia at least, restricted to older adults. Furthermore, inpatient mortality rates are much lower in younger compared with older inpatient groups. The FI predicts adverse inpatient outcomes such as delirium and in-hospital falls in older adults^15^ and it would be of interest to explore whether this is also the case in younger adults. Larger sample sizes may also enable further age disaggregation, which may yield important insights.

Another limitation is that socioeconomic factors, including educational level, employment status, income and housing status, were not collected by the assessment instrument. Consequently, the prevalence of these factors in this sample could not be examined. While some data regarding ethnicity, cultural and linguistic diversity and social support were collected, the modest sample size meant case numbers among younger adults were small. Socioeconomic vulnerabilities have been associated with specific patterns of multimorbidity in younger adults in the community,^16^ but they have not been examined in relation to frailty in this age group in community or hospital settings. Future studies may aim to explore the relationship between socioeconomic status and frailty in a larger sample of younger adult inpatients. It would then be of interest to compare and contrast findings with those of a study conducted in younger adult community-dwellers.

Overall, this study showed that frailty is prevalent in younger patients admitted to acute care and is associated with prolonged hospitalization. Mood and behavioural symptoms, other health symptoms, nutrition and pain are key issues for this patient group. This study was a critical first step towards establishing an understanding of frailty in younger hospitalized adults.
